# Relationship Between Team Identification and Consumption Behaviors: Using a Meta-Analysis

**DOI:** 10.3389/fpsyg.2022.869275

**Published:** 2022-05-13

**Authors:** Hyungil Kwon, Do Young Pyun, Seung Hyun Lim

**Affiliations:** ^1^Department of Physical Education, Chung-Ang University, Seoul, South Korea; ^2^School of Sport, Exercise and Health Sciences, Loughborough University, Loughborough, United Kingdom

**Keywords:** team identification, sport consumption, intention to attend sport games, intention to purchase licensed merchandise, meta-analysis

## Abstract

The purpose of this study was to conduct a comprehensive literature review and a meta-analysis on team identification, particularly its relation to two outcome variables: intention to attend games and intention to purchase licensed merchandise. Past literature was searched through several electronic databases, including SPORTDiscus, using relevant keywords. Thirteen studies for team identification and intention to attend sport games and nine studies for team identification and intention to purchase licensed merchandise meeting all selection criteria were identified. A random-effect model was used to conduct a meta-analysis. Funnel plot and Egger's test were conducted to test publication bias, followed by fail-safe N to examine the stability of the estimates. The results showed that the total effect size values for the relationships between team identification and intentions to attend games and to purchase licensed merchandise were 0.39 and 0.42, respectively, which indicates a medium-large effect size. The study confirms the predictive power of team identification when it pertains to sport consumption behaviors and provides insights into practical implications in utilizing team identification as a potential driver of promoting consumer behaviors by the way of achieving marketing objectives.

## Introduction

Since the term team identification first appeared in sports literature (Gantz, 1981), many empirical studies have been produced and accumulated. Team identification, which refers to fans' psychological connections to a team (Wann, 2002), has been popularly examined by scholars and practitioners due to its predictability on sport consumers' behavior, such as game attendance and licensed merchandise consumption. For example, Lee et al. (2013) reported that the Pearson correlation coefficient between team identification and licensed merchandise purchasing intention among collegiate game attendees was as high as 0.82. This indicates that more than 67% of the variance in the purchase intention of licensed merchandise is explained by team identification. In another study by Theodorakis and Wann (2008), team identification explained 36% of the variance of the intention to attend a sport game. As such, team identification has been employed in numerous empirical studies and has consistently shown statistically significant influence on sport consumers' behaviors which are directly connected to the revenues of sport teams (Fink et al., 2002).

Although most of the published empirical studies have shown statistically significant correlations in the relationships between team identification and sport consumer behaviors, their findings are fragile. The correlation values actually vary arbitrarily even though resultant behaviors are identical. As noted earlier, Lee et al. (2013) reported a 0.82 correlation value between team identification and licensed merchandise consumption. However, 1 year later, Kwon and Kwak (2014) reported the correlation between the same variables to be 0.21. Although both correlations were statistically significant at the alpha level of 0.05, many empirical studies in sport marketing reported heterogeneous magnitude of correlation coefficients between these two variables across different contexts or populations. There is a need to collate empirical findings in this subject area from a wider range of previous sport marketing literature and provide sound evidence in support of the fact that team identification influences attendance and purchase intention.

Therefore, after accumulating a sufficient number of empirical studies on team identification, a meta-analysis on the relationship between team identification and sport consumers' behaviors is necessary to provide a comprehensive overview of the predictability of team identification on sport consumers' behaviors, such as game-attending behavior and licensed merchandise consumption. According to Glass (1976), a meta-analysis refers to “the statistical analysis of a large collection of analysis results from individual studies for the purpose of integrating the findings” (p. 3). The advantage of a meta-analysis is evidenced by its process of combining the summary results rather than raw data from existing studies and focusing on the size of treatment effect and relationship strength, and not just statistical significance (Brockwell and Gordon, 2001). Thus, through the process of a meta-analysis, the current study should be able to provide the strength of the correlations between (a) team identification and intention to attend sport games and (b) team identification and intention to purchase licensed merchandise by synthesizing and quantifying the previous findings. This systematic analysis would overcome the shortcomings of the individual studies, particularly when reporting different magnitudes in the strength of correlation coefficients between the aforementioned variables, and would provide a better understanding of how both links are associated.

Therefore, the purpose of the study is two-fold. First, using the advanced meta-analytic techniques, the study sought to systematically review and analyze the effect sizes of both correlations between team identification and its resultant behaviors of game attendance and licensed merchandise consumption. Second, the study compared the magnitude of the correlations in the paths to see if, to which resultant behaviors, team identification has more predictive power.

### Measuring Team Identification and Its Theoretical Ground

Since Gantz (1981) first empirically examined the relationship between team identification and sport fans' TV viewership, it has attracted many scholars' interests, as it has been evidenced that team identification explains a significant amount of variance in sport consumers' behaviors. The surge of interest in team identification can be witnessed in the number of studies published in diverse academic journals. Most of the studies on team identification have been published in key sport management journals, such as the Journal of Sport Management (e.g., Heere and James, 2007a; Lock et al., 2012, 2014) and Sport Management Review (e.g., Kwon et al., 2005; Heere and James, 2007b; Lock and Funk, 2016). It is probably because sports management is a business-oriented discipline and needs to have its own concepts and variables that explain business aspects of professional and collegiate sport contexts.

Although Gantz (1981) initiated the research endeavor on team identification and consumption behaviors among sport fans, Wann and his colleagues began developing systematic programs for the investigation purpose in the 1990's (e.g., Wann and Branscombe, 1990, 1993; Wann et al., 1999). Although Wann and Branscombe (1990) first published a research article on the role of identification within a group, a measurement tool was not available until 1993 (Wann and Branscombe, 1993). Wann and Branscombe (1993) developed a seven-item scale labeled the Sport Spectator Identity Scale. Although there was a criticism on this scale that it lacked theoretical ground, it has been one of the most frequently used scales in empirical studies (Lock and Heere, 2017). In addition to the Sport Spectator Identity Scale, Lock and Heere (2017) indicated that the Team Identification Index (Trail et al., 2003) has also been utilized in many studies. Although the two scales (Wann and Branscombe, 1993; Trail et al., 2003) were the most popular measures used in the previous studies on team identification, the scales have been criticized for their atheoretical nature (Lock and Heere, 2017).

Although theoretical soundness of team identification is not a major concern of the current study, it needs to be clarified that all the empirical studies included in the meta-analyses were at least on the same theoretical ground. With regards to a theoretical ground of team identification, an interesting study was published by Lock and Heere (2017). They argued that two different theories of social identity theory (Tajfel and Turner, 1979) and identity theory (Stryker, 1968) were used in previous studies as the ground for team identification. Social identity theory explains an individual's self in relation to the social group that they belong to. For instance, if a person is a fan of Liverpool FC, they categorize other fans, along with them, into a social category of “the Reds” and form an in-group. This automatically forms an out-group who are “not the Reds”. In the process of self-categorization, the fan sees themselves as a member of the fan group, which determines their social identity. On the other hand, identity theory focuses on the role of an individual in a social structure (Stets and Burke, 2000). For instance, identity theory explains that the individual's self depends on the roles that they perform in a social structure rather than a subgroup of “fans”. Thus, social identity theory focuses on an individual's “being,” whereas identity theory focuses on an individual's “doing”.

Lock and Heere (2017), along with Hogg et al. (1995), emphasized the differences between social identity theory and identity theory. Particularly, Lock and Heere (2017) followed the idea of Hogg et al. (1995), where the two theories differed in the “level of analysis”. Specifically, Hogg et al. (1995) differentiated two theories by addressing that identity theory does not have a strong psychological background, thus failing to explain the generative cognitive process firmly. This is mainly because the social identity theory provides an explanation on cognitive processes of depersonalization and self-categorization, whereas the identity theory just labels one person with a noun (e.g., soldier, nurse, etc.), as each person belongs to a social category (Hogg et al., 1995). Furthermore, Hogg et al. (1995) highlighted the strength of social identity theory with a belief that it seeks to elaborate the psychological level of analysis, along with the sociological level, in a systematic manner.

However, Lock and Heere (2017), citing Hogg et al. (1995) and Stets and Burke (2000), argued that the differences in the “level of analysis” are “team fan (identity theory)” and “a sport team (social identity theory)” because identity theory focuses on an individual fan but social identity theory is concerned with a team. Their interpretation of a “different level of analysis” seems to show a serious conceptual gap from what Hogg et al. (1995) noted. Because Hogg et al. (1995) noted that both theories have a strong conceptual base in a multi-dimensional aspect and dynamic self that plays as a mediator in the linkage between social structure and individual behavior, both individual and team levels may not be considered as the core concept of “different level of analysis”. What is really meant by “different level of analysis” is that social identity theory includes a psychological level of analysis, whereas identity theory does not. Ravasi and van Recom (2003) also supported this distinction by adding that social identity theory is more concerned with how individuals perceive, while identity theory emphasizes an organizational level or even a society level. To sum up, social identity theory focuses on an individual self rather than the social category to which an individual belongs to.

Although Lock and Heere (2017) followed the interpretation of Stets and Burke (2000) when they explained the concept of “level of analysis,” Stets and Burke (2000) consistently argued that social identity theory and identity theory shared substantial similarities and overlap. Their argument is, basically, that “what I am” cannot be separated from “what I do”. Although the two theories are different in origins (i.e., identity theory from sociology and social identity theory from social psychology) and languages (e.g., “identification” in identity theory and “self-categorization” in social identity theory), they are similar in nature, and the difference is derived from a matter of emphasis rather than a matter of kind (Stets and Burke, 2000).

Lock and Heere (2017) argument that team identification has two different theoretical backgrounds provides conceptual advancement in the use of team identification as a predictor of consumption behaviors. However, the resultant behavior of a fan (e.g., attending a sport game or purchasing licensed merchandise) cannot be attributed to one specific theory, that is, identity theory or social identity theory. While a fan could purchase a licensed club jersey as a role of a fan (identity theory), at the same time, they could purchase it to represent their social identity as a fan of the team (social identity theory). In the same vein, a fan attends a game and supports their team as a role of a fan (identity theory), as well as a fan group member (e.g., social identity theory). Thus, the theoretical differentiation would result in limited applicability, particularly when it pertains to fan behavior. In addition, Hogg et al. (1995) noted that the different level of analysis involves the psychological cognitive process. However, the focal aspect of this meta-analysis is not regarding how they became a fan of a team but the behaviors of fans, which is not the case with the “different level of analysis”.

From the perspective of the relationship between cognitive algorithmic processes and behavioral choices (e.g., Andronie et al., 2021; Rydell and Kucera, 2021), the linkages between group (team) identification and consumption behaviors are also underpinned. Rydell and Kucera (2021) conducted a systematic review on consumer adoption of mobile shopping apps and concluded that consumer engagement and willingness to adopt mobile commerce apps significantly influenced their purchase decision-making process. With a similar conceptual disposition, Andronie et al. (2021) explained how consumer shopping and dietary behaviors were determined by their cognitive attitudes during the COVID-19 pandemic.

Thus, in the current study, it would be safer to deem that team identification has been investigated on an identical theoretical foundation and to hypothesize that team identification is a significant predictor of both intentions to purchase licensed merchandise and attend a game. With this proposition, the current study reviewed the existing literature that examined the impact of team identification on attendance and merchandise purchase behaviors and calculated effect sizes from the correlations between the relationships to determine the predictability of team identification.

## Methods

### Literature Search

In an effort to locate all relevant research, an extensive search of the literature was conducted. The main electronic database for the current study was SPORTDiscus, which is known to be the leading bibliographic database for sports research. In addition, other popular databases such as Web of Science, Scopus, ScienceDirect, EThOS, PsycARTICLES, PsycINFO, ProQuest, and Google Scholar were also scrutinized using the keywords team identification, identification, purchase intention, attendance intention, consumption intention, behavioral intention, fan behavior, consumption behavior, team-licensed merchandise, and team merchandise. Any relevant articles not reporting their correlation results were not included in this assessment.

For the relationship between team identification and intention to attend sport games, a total of 13 studies were identified. Among those, 11 articles were published in academic journals, and two were unpublished doctoral dissertations. For the relationship between team identification and intention to purchase licensed merchandise, a total of nine studies were selected, and all of them were journal articles. Some of the studies reported multiple correlations in a study. These multiple correlations were included in the analysis only if each correlation represented a unique combination to team identification (Damanpour, 1991). For example, Shapiro et al. (2013) reported three different Pearson correlation coefficients for the intention to attend future games for football, men's basketball, and women's basketball. The Pearson correlation coefficients were 0.44, 0.26, and 0.10 (as they appeared in their study), respectively. However, the research participants were the season ticket holders and student ticket holders of football games. Thus, the current study only used the Pearson correlation coefficient of the football game, which was 0.44.

Among the studies selected for the current meta-analysis, the Sport Spectator Identification Scale (Wann and Branscombe, 1993), Sport Fandom Questionnaire (Wann, 2002), Team Identification Index (Trail and James, 2001), Points of Attachment Index (Robinson and Trail, 2005), Sport Spectator Identification Scale (Mael and Ashforth, 1992; Madrigal, 2001), organizational identification scale, and Bergami and Bagozzi, 2000 cognitive identification scale were used to measure team identification. Damanpour (1991) investigated the correlations between organizational innovation and other variables, such as functional differentiation, professionalism, and so on, by performing a meta-analysis. In the study, Damanpour (1991) defined organizational innovation and used different scales of organizational innovation in the meta-analysis.

### Analytic Procedures

To maintain good quality in a meta-analysis, clear standards in coding should be shared among coders (Schmitt et al., 1991). For this study, two independent coders entered relevant information in an Excel spreadsheet. In the Excel spreadsheet, each coder is instructed to enter data regarding the authors of the study, year of publication, sample size, correlations, and publication types (i.e., journal article or thesis/dissertation). The coders worked separately. Following the procedure of Bullock and Svyantek (1985), the coders discussed inconsistencies until the coders reached an agreement.

As a random-effect model was used to conduct a meta-analysis, initially *Q*-values and *I*^2^ statistics were calculated to determine the heterogeneity across the collected studies. Once the heterogeneity was confirmed (*Q*-value is significant at *p* = 0.05), the meta-analysis was conducted by computing the average weighted mean correlations between team identification and both intentions (c.f., Hedges and Olkin, 1985). The Cohen's effect size measures were used in the meta-analysis, indicating 0.10, 0.30, and 0.50 as small, medium, and large effects, respectively. Next, the funnel plot and Egger's test were carried out to test publication bias (Egger et al., 1997), followed by the stability test for the meta-analysis using fail-safe N (Rosenthal, 1979). Finally, a group comparison analysis was conducted to determine whether the computed correlations between the two relations were statistically different or not.

## Results

A summary of the identified studies (*N* = 22) is presented in Tables 1, 2. There were 13 studies with regard to the relationship between team identification and intention to attend. Nine studies examined the relationship between team identification and intention to purchase. A majority of the studies used college students as their research samples. Two studies examining team identification and intention to attend sport games were doctoral dissertations (see Tables 1, 2 for more details).

**Table 1:** Pearson correlation coefficients (r) between team identification and intention to attend sport games.

**No**	**Authors**	***r***	**N**	**Sample**	**Publication type**
1	Melnick and Wann (2011)	0.32	163	Students	Journal article
2	Gray and Wert-Gray (2012)	0.50	300	Students	Journal article
3	Park and Dittmore (2014)	0.34	146	Students	Journal article
4	Theodorakis et al. (2017)	0.41	259	Students	Journal article
5	Parry et al. (2014)	0.24	252	Students	Journal article
6	Theodorakis and Wann (2008)	0.60	351	Students	Journal article
7	Williams et al. (2012)	0.12	492	Generic	Journal article
8	Swanson et al. (2003)	0.44	537	Students	Journal article
9	Shapiro et al. (2013)	0.44	1091	Student, generic	Journal article
10	Drayer et al. (2018)	0.15	260	Generic	Journal article
11	Chen (2009)	0.53	548	Students	Master thesis
12	Chen (2013)	0.38	478	Students	Doctoral dissertation
13	Tan (2011)	0.50	348	Students	Doctoral dissertation

**Table 2:** Studies that included Pearson r between team identification and intention to purchase licensed merchandise.

**No**	**Authors**	***r***	**N**	**Sample**	**Publication type**
1	Matsuoka et al. (2003)	0.41	1256	Generic	Journal article
2	Carlson et al. (2009)	0.39	162	Students	Journal article
3	Gray and Wert-Gray (2012)	0.50	300	Students	Journal article
4	Lee and Kang (2015)	0.51	259	Generic	Journal article
5	Kwon and Kwak (2014)	0.21	203	Students, alumni	Journal article
6	Shapiro et al. (2013)	0.25	1091	Generic	Journal article
7	Lee et al. (2013)	0.82	358	Students	Journal article
8	Kwon and Armstrong (2004)	0.35	200	Students	Journal article
9	Lee and Trail (2012)	0.28	736	Students	Journal article

To understand the heterogeneity of the correlations included in the meta-analysis, Q-values (to see whether a true effect size varied among the studies) and *I*^2^ estimates (to see the magnitude of heterogeneity when a Q-value is significant) were calculated. *Q*-values were 120.32 (*p* < 0.001) for the relationship between team identification and intention to attend sport games and 255.06 (*p* < 0.001) for the relationship between team identification and intention to purchase licensed merchandise. The *I*^2^ values were 90.00 and 96.83, respectively, for intention to attend sport games and for intention to purchase licensed merchandise. *I*^2^ is the proportion of total variation in estimates stemming from heterogeneity (Higgins and Thomson, 2002). Based on the heterogeneity across the values of the correlations, a random-effect model was recommended to analyze the studies.

The results of the meta-analyses indicated that the comprehensive correlation between team identification and intention to attend was 0.39 (*z* = 31.34, *p* < 0.001), with confidence intervals of 0.37 and 0.42. The correlation between team identification and intention to purchase was 0.42 (*z* = 7.43, *p* < 0.001), with the confidence interval of 0.30 and 0.56 (see Table 3). The correlations were considered to be medium to large based on (Cohen, 2013) suggested criteria.

**Table 3:** Meta-analysis results of the relationship.

**Group**	**No. of studies**	**Point estimates**	**Test of null**		**95% CI**	**Heterogeneity**	
			***z***	***p***		**Q-value**	***p***
1^a^	13	0.39	31.35	<0.001	(0.37, 0.42)		
2^b^	9	0.42	7.43	<0.001	(0.30, 0.56)		
Total between						0.72	0.40

a *Team ID <-> Intention to attend sport games*.

b *Team ID <-> Intention to purchase licensed merchandise*.

**Figure 1:**
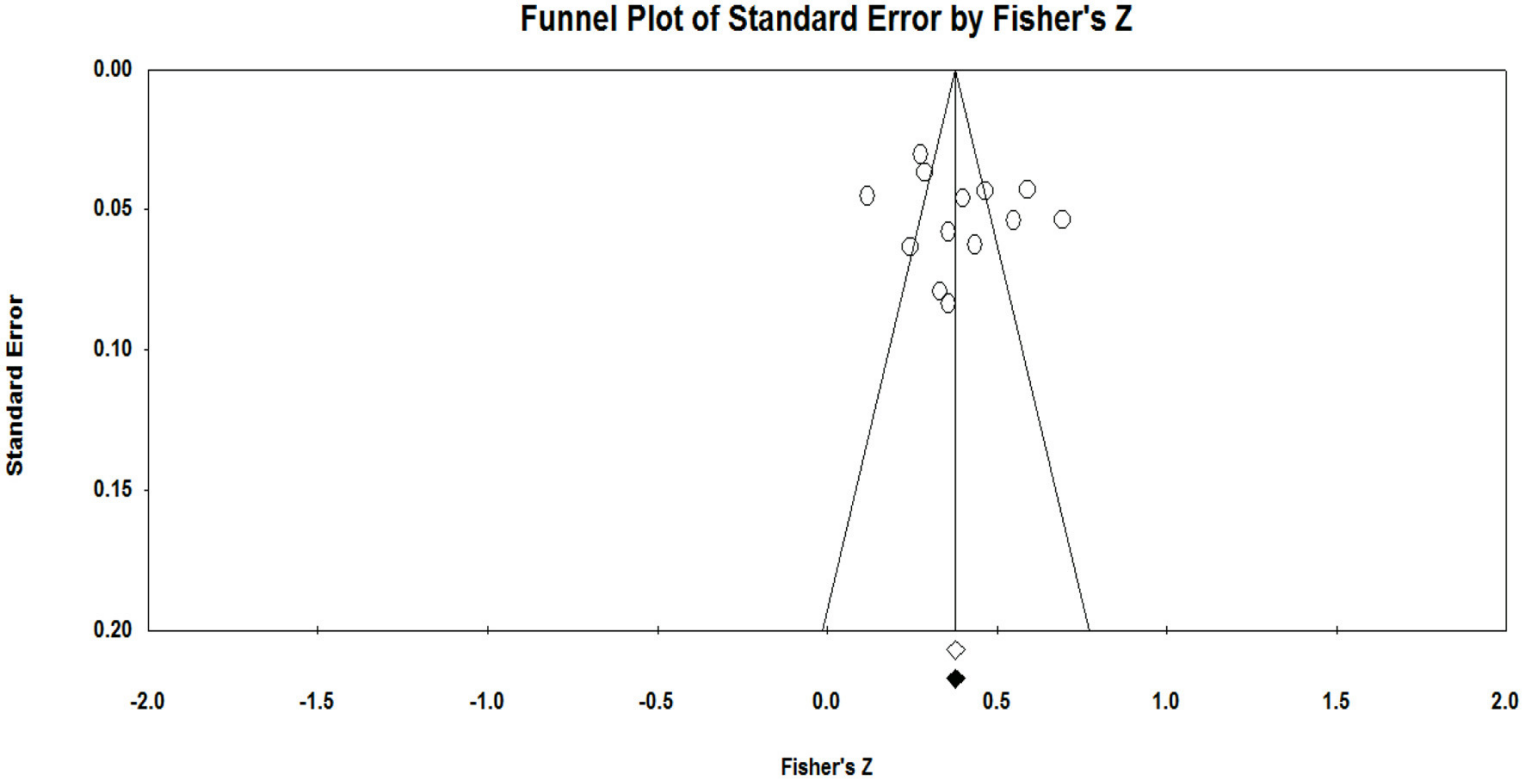
Funnel plot of team identification and intention to attend sport games.

**Figure 2:**
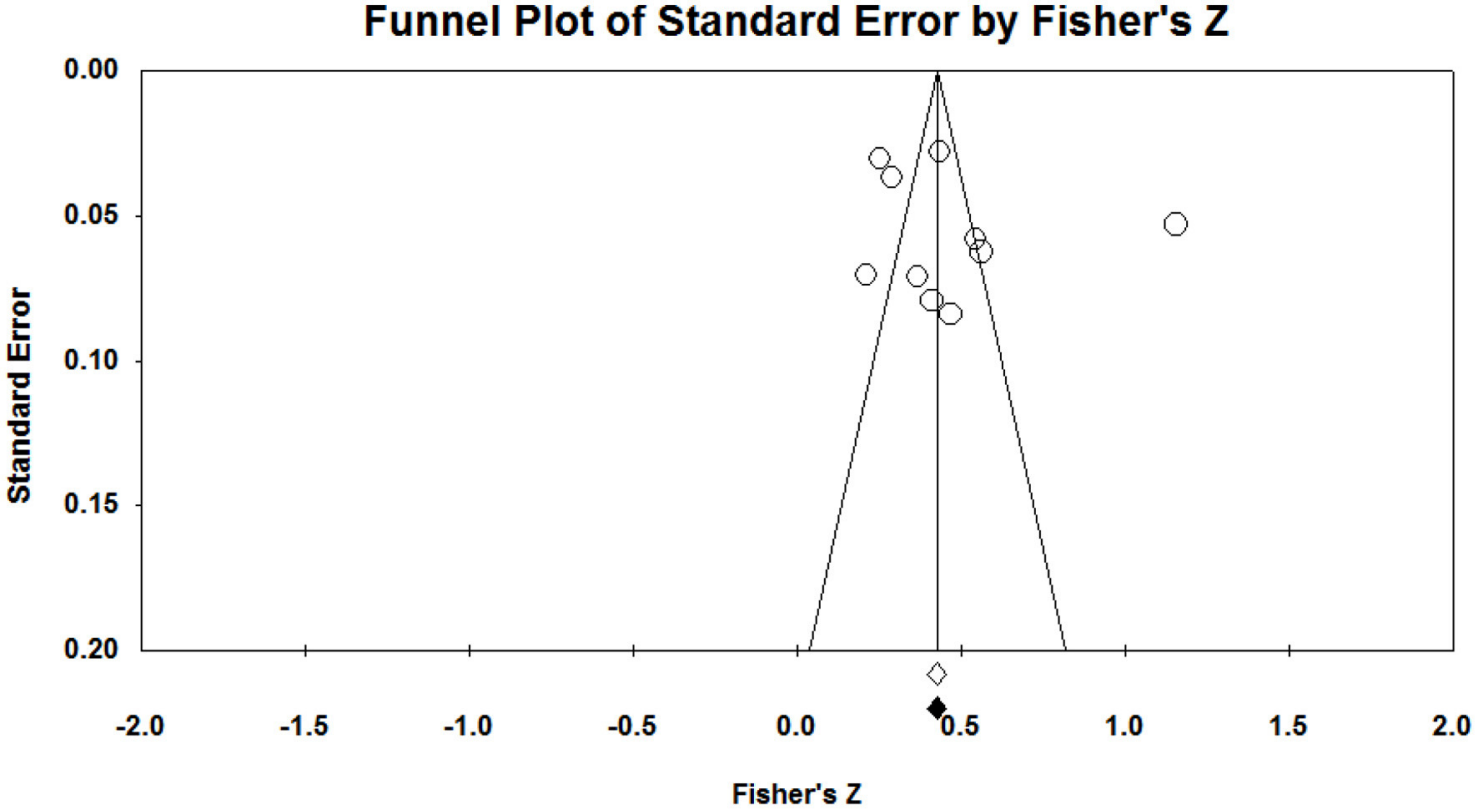
Funnel plot of team identification and intention to purchase licensed merchandise.

In an effort to examine the publication bias, two analyses of the funnel plot and Egger's test for publication bias were conducted (Egger et al., 1997). The results of the funnel plot for team identification and intention to attend sport games are presented in Figure 1. The funnel plot had a relatively even number of studies on the left and right sides of the funnel. Likewise, Egger's test was not rejected with a *p*-value of 0.46 in the two-tailed analysis. The 95% confidence interval ranged from −4.95 to 10.23, which included “0,” thus supporting the hypothesis testing. Thus, it can be concluded that the analysis did not have a statistically significant publication bias. Figure 2 shows the results of the funnel plot and Egger's test for team identification and intention to purchase licensed merchandise. The funnel plot had a relatively similar number of studies on the left and right sides of the graph, although there was one study quite out-lied to the right side. Accordingly, Egger's test was not rejected with a *p*-value of 0.45 in the two-tailed analysis. The 95% confidence interval ranged between −8.28 and 16.80, which included “0,” thus supporting the hypothesis testing. The trim-and-fill method was also utilized to provide a summary effect adjusted for publication bias. The analysis recommended no additional study, and there was no change in the point estimate. Thus, the analysis did not show any statistically significant publication biases.

Next, Rosenthal's fail-safe N was calculated to assess the stability of the meta-analytic results (Rosenthal, 1979). Rosenthal's fail-safe N considers a number of new unpublished or unretrieved non-significant studies that would change the results of the meta-analysis (i.e., an alpha level of the current study was set as 0.05). The results of Rosenthal's fail-safe N for the relationship between team identification and intention to attend sport games was 3,018, and the one for the relationship between team identification and intention to purchase licensed merchandise was 1,877. The validity of the N can be achieved when N is greater than its tolerance level, which is calculated using the formula of *t* = 5*k* + 10. In the case of the intention to attend sport games, the tolerance level was 75, and the level for the licensed merchandise consumption was 55. In both cases, the calculated N value was much greater than the tolerance level. Thus, the results of the meta-analysis of the study are deemed quite stable.

Additional group comparison analysis was performed to see if the comprehensive correlations between team identification and both behavior intentions were statistically different. The results of the group analysis are presented in Table 3. The results of the analysis indicated that the comprehensive correlations in the two groups were statistically identical (*Q*-value = 0.72, *p* = 0.40). Thus, it can be concluded that the predictive power that team identification has toward attendance intention is statistically the same with respect to licensed merchandise consumption intention.

## Discussion

Since Gantz (1981) first introduced the term player/team identification to explain sport fans' television audience behavior, many studies have employed the concept of team identification in their empirical tests with different methods and populations. Although, as Lock and Heere (2017) highlighted, early studies of team identification failed to build a robust theoretical foundation of team identification, recent studies have been conducted on the sound theoretical grounds which are based on either identity theory or social identity theory (e.g., Kwon and Armstrong, 2002; Matsuoka et al., 2003; Carlson et al., 2009).

Among many known variables in sport consumer behavior research, team identification has relatively attracted a greater amount of interest from scholars, mainly due to its predictive power. Team identification has been known to be a strong predictor of game-attending behavior, licensed merchandise consumption, and other fan behaviors, such as aggression (e.g., Wann et al., 1999) and satisfaction (e.g., Matsuoka et al., 2003; Gray and Wert-Gray, 2012). As confirmed by this study, the comprehensive correlation value between team identification and intention to attend sport games was 0.39, and the value between team identification and intention to purchase licensed merchandise was 0.42. Cohen (2013) defined the Cohen's *d* values of 0.20, 0.50, and 0.80 as small, medium, and large effect sizes for one-sample *t*-test, respectively, and their corresponding values of *r*s for Pearson correlation are 0.10, 0.30, and 0.50. Thus, the correlation values found in this study seem to indicate medium to large effects, quantifying the predictive accuracy of the variable. This finding is consistent with the previous arguments that consumer engagement (e.g., a sense of belongingness) with a team explains their purchase behavior for goods/services associated with the team to satisfy the need for self-identity rather than the functional need (e.g., Kwon and Armstrong, 2002; Lee and Trail, 2012). Furthermore, the results of the study show that team identification has a stronger correlation with the licensed merchandise consumption, albeit it was not statistically differentiated.

An interesting finding from the study is that the effect sizes varied among the empirical studies on team identification. For example, the correlation between team identification and intention to attend sport game ranged from 0.12 (Williams et al., 2012) to 0.60 (Theodorakis and Wann, 2008), showing considerable variation. Likewise, the correlations between team identification and licensed merchandise consumption were also widely distributed from 0.21 (Kwon and Kwak, 2014) to 0.82 (Lee et al., 2013). Moreover, a similar study (Riketta, 2005) was conducted on the existing organizational behavior studies with a meta-analysis. In the study, the comprehensive correlations between organizational identification and a wide range of work-related attitudes, behaviors, and context variables were examined. The study revealed similar results that the mean correlations with work-related attitudes (attitudinal organizational commitment, satisfaction, and involvement), context variables (job scope/challenge and organizational prestige), and behaviors (in-role and extra-role performance) were large, medium-large, and small to medium, respectively (Riketta, 2005). Therefore, the conclusions interpreted from the current results may not be applicable to any single setting and measure.

As mentioned in the Introduction section, a meta-analysis on the predictive nature of team identification was needed because many empirical studies reported heterogeneous levels of the findings across team identification research. In this study, we attempted to identify a possible reason to explain this heterogeneity. First, a subgroup analysis using publication type as a moderator was conducted. However, the analysis found that publication type was not a statistically significant moderator. There are other possibilities explaining the heterogeneity in the effect sizes in the empirical studies. For instance, the effects of team identification may vary depending on the type of sport. However, a subgroup analysis of the type of sport was not considered in this study because similar numbers of studies were not available for each sport. Out of 13 studies for team identification and game attendance intention, six studies used generic sport, while the other studies were related to American football (4), basketball (2), baseball (1), and ice hockey (1). The studies for team identification and merchandise purchase intentions also showed a similar pattern; four studies used generic sport, followed by American football (2), basketball (2), and soccer (1). Thus, it is not expected that there would be a possible moderating effect of sport type on the relationships. Another possibility explaining the heterogeneity in the effect size is the use of different measurements to measure the resultant behavior. For example, Shapiro et al. (2013) measured the purchase intention of licensed merchandise in dollars, whereas Gray and Wert-Gray (2012) used a five-point Likert scale to measure the same behavioral intention.

One of the main purposes of a meta-analysis is to aggregate data across the studies in an effort to comprehensively quantify them. Although a meta-analysis applies objective formulas to deal with a large number of studies, there would be a couple of limitations that could bias the estimates. One is publication bias, which could be derived from the possibility that any studies failing to reveal significant findings (e.g., correlations) may not have chances to be published and included in the analysis (Esterhuizen and Thabane, 2016). On the other hand, some published studies showing significant effects could be missed during the search process and were not included in the data analytics procedures. Both cases could lead to underestimation or overestimation of the true effects. Generally, a study with a small sample size tends to produce a larger effect size. Thus, a possible publication bias could be gauged by comparing a sample size and an effect size in a study. This study used a funnel plot and Egger's analysis to test publication bias. First, the funnel plots in Figures 1, 2 show that there are relatively similar numbers of the studies on the left and the right sides. As this scatterplot of the estimates shows eyeball evidence (usually symmetrical about the mean effect size), the Egger's tests were followed up to conduct statistical testing, which is considered to be a sound assessment technique (Egger et al., 1997). This technique is particularly useful for smaller studies that are analyzed with less methodological rigor for a funnel plot (Egger et al., 1997). Both tests did not show possible publication biases.

However, Peters et al. (2006) addressed a concern regarding the Egger's regression test due to its high type I error. Extra caution is necessary for detecting funnel plot symmetry. In this context, to increase the stability of a meta-analysis, fail-safe *N*, which estimates how minimally missing papers could reduce the total effect sizes rather than treating them as a zero (Rosenthal, 1979), was calculated in this study. While Rosenthal's fail-safe *N* value supports the validity of the current effect sizes, this technique has been challenged because an estimate of fail-safe *N* is highly influenced by a mean intervention effect, as the intervention effects of any omitted studies are considered to be zero (Sterne et al., 2008). The “trim-and-fill” would be an alternative method to calculate an adjusted mean intervention effect by (a) trimming studies which could result in funnel plot asymmetry, (b) estimating the center of the funnel, and (c) filling omitted studies and their missing counterparts around the estimated center (Sterne et al., 2008). Overall, it is clearly evidenced that publication biases, as a cause of the small-study effect in a meta-analysis, tend to overestimate an intervention effect (Sterne et al., 2008). The publication bias tests used in the present study confirm that a small-study effect was not detected in this meta-analysis.

## Conclusion

To sum up, this study presents a more comprehensive literature review and a meta-analysis of empirical research on team identification and its relationship with both intention to attend sport games and intention to purchase licensed merchandise. More specifically, the study is purported to conduct empirical assessments of how robust the predictive accuracy of team identification is over different sports, measurements, populations, and settings. Our research reveals the medium-large size effects, showing sufficient magnitudes of the relationships. It is concluded that the impacts of team identification on fan behaviors in the sport marketing literature are consequential.

While this study presents strong support for the team identification and consumption behavior linkage, it does not address a possible change in the consumers' behavior patterns through the COVID-10 pandemic (Rydell and Kucera, 2021; Valaskova et al., 2021; Watson and Cug, 2021). The crisis may influence people's cognition or sentiment, resulting in panic buying and stockpiling behaviors (e.g., Birtus and Lazaroiu, 2021), or shrink their consumption for certain industries due to social distancing restrictions, leading to the cancellation or suspension of spectator sporting events (Skinner and Smith, 2021). Sport consumers have adopted more sustainable or other forms of consumption patterns (e.g., home fitness or Sports). Thus, it is also worth exploring how the COVID-19 pandemic has impacted the role of team identification in predicting traditional consumption behaviors. Understanding sport fans' future behaviors during the pandemic can help sport marketers cope with the uncertainty they experience by implementing necessary crisis and emergence marketing strategies wisely and decisively.

## Data Availability Statement

The original contributions presented in the study are included in the article/supplementary material, further inquiries can be directed to the corresponding author.

## Author Contributions

HK is the leading author of the manuscript. DP collected data and wrote a significant part of the paper. SL mainly collected the data for the meta analysis. All authors contributed to the article and approved the submitted version.

## Conflict of Interest

The authors declare that the research was conducted in the absence of any commercial or financial relationships that could be construed as a potential conflict of interest.

## Publisher's Note

All claims expressed in this article are solely those of the authors and do not necessarily represent those of their affiliated organizations, or those of the publisher, the editors and the reviewers. Any product that may be evaluated in this article, or claim that may be made by its manufacturer, is not guaranteed or endorsed by the publisher.
